# Breast self-examination practice and associated factors among women of reproductive age in southeast Ethiopia

**DOI:** 10.3389/fonc.2023.1176022

**Published:** 2023-06-08

**Authors:** Abduljewad Hussen, Musa Kumbi, Jemal Bedewi, Abate Lette, Shemsu Nuriye

**Affiliations:** ^1^ Department of Public Health, Goba Referral Hospital, Madda Walabu University, Goba, Ethiopia; ^2^ Department of Public Health, Madda Walabu University, Shashemene, Ethiopia; ^3^ Department of Public Health, College of Medicine and Health Science, Wolkite University, Wolkite, Ethiopia; ^4^ Department of Public Health, College of Medicine and Health Science, Wolayta Sodo University, Sodo, Ethiopia

**Keywords:** reproductive age group, breast self-examination, breast cancer screening, Ethiopia, gurage zone

## Abstract

Regular breast self-examination is the most cost-effective method for early detection of breast cancer, especially in resource-limited countries. However, breast self-examination practice was low among reproductive-age women.

**Objective:**

This study aims to evaluate breast self-examination practice and associated factors among women of reproductive age in southeast Ethiopia.

**Methods:**

A convergent parallel mixed-methods study design was conducted on 836 women of reproductive age. An interviewer-administered questionnaire was used for the quantitative part of the study and supplemented by focus group discussions. A database was created using Epi-info version 3.5.3, and analyzed using SPSS version 20. Bivariate and multivariable logistic regressions were done to examine the effect of explanatory variables. Variables with *p*-value <0.05 during multivariable logistic regressions were considered as significantly associated with the dependent variable. Thematic data analysis was carried out for the qualitative study.

**Result:**

Out of 836 total participants, only 20.7% had ever heard about breast self-examination practice. Also, only 13.2% of the mothers had practiced breast self-examinations. Even though the majority of the participants during focused group discussions had knowledge about breast cancer screening, most of them stated that breast self-examination was not practiced among them. Maternal age, mother’s level of education, and previous history of breast examination by health professionals were significant predictors of breast self-examination practices.

**Conclusion:**

This study reported a low prevalence of breast self-examination practice. Therefore, enhancing women’s education and encouraging breast examination by health professionals are essential to increase the proportion of women performing breast self-examination.

## Introduction

Breast cancer (BC) is typically asymptomatic when the tumor is small and easily treated. Therefore, it is very essential for women to follow suggested screening rules in detecting BC in the early stage (1). Before BC develops to a size that can be felt, the most common physical sign is a painless lump or swelling until the original breast tumor itself is large enough to be felt (2–4). Other common signs and symptoms include breast pain or heaviness, persistent changes in the breast (swelling, thickening, or redness of the breast’s skin), nipple abnormalities (spontaneous bloody discharge), erosion, inversion, and tenderness (1, 5).

Age is the most important factor for BC risk in women. It will affect women in their most productive years of life. More breast cancer cases are identified in developing countries than those in developed countries among women of reproductive age (15–49 years) (6). The other risk factors for BC are inherited mutations in susceptible genes; a personal or family history of BC; high breast tissue densities; high dose radiations to the chest resulting from medical procedures (7); reproductive factors that increase risks (early start of menstrual periods and/or end later in life); not ever having children; prolonged use of oral contraceptives; and having a child after the age of 30. Some other factors that increase risk include being overweight or obese after menopause, use of menopausal hormone therapy (combined), physical inactivity, and consumption of alcohol (8).

The most imperative strategies in achieving early detection of BC are mammography and physical examination of the breasts by qualified health workers, or clinical breast examination (CBE) and breast self-examination (BSE) (6, 9, 10). BSE has been broadly recommended as a comparatively simple, non-offensive, non-harmful, and cost-free screening technique when comparing to other types of screening approaches for breast cancer (11, 12). It is a technique established for the purpose of assessing cancer; a woman uses her hands to systematically examine her breasts and the immediate areas for unfamiliar lumps and shape changes. Usually done on a fixed monthly basis, the same technique is used each time, ensuring that all areas of the breast are sensed and checked thoroughly.

In 2018, breast cancer represents approximately 24.2% of all new cancer cases and 15% of 4.2 million deaths due to cancer globally, of which sub-Saharan Africa accounts for 8.1% of breast cancer cases and 11.8% of breast cancer deaths. It is the most prevalent cancer in developing countries (6, 9, 13). In Ethiopia, BC is typically a fatal disease among women with a 24.4% prevalence rate (6, 14). Early detection makes the disease easier to treat. Some authors suggest that BSE does not significantly reduce the breast cancer mortality, but the awareness among women about their breasts provides them with knowledge to seek medical help early in order to improve the prognosis (15, 16). BSE is a recommended diagnostic method and an important technique in the prevention of breast cancer, especially in resource-limited countries (6, 14, 15). However, stigma towards cancer; poor knowledge of BC related to signs, symptoms, and its treatability; and system overload continue to account for delays in reaching care (17). Ethiopian women are usually alerted in the late stage of the disease (10, 18). In Ethiopia, reproductive-age women know little about BSE (6). Therefore, this study was designed to identify the prevalence of BSE and associated factors among reproductive-age women in Bale Zone, southeast Ethiopia.

## Materials and methods

### Study design, area, and period

A convergent parallel mixed-methods study design was conducted from 1 March 2017 to 30 May 2017 in Bale Zone. Robe town is the capital city of Bale Zone, which is 430 km from Addis Ababa, the capital city of Ethiopia. The Bale Zone has 20 districts with farming communities predominating. The study was conducted in Ginnir, Sinana, and Madda Walabu districts, which are located at 560 km, 430 km, and 630 km southeast of Addis Ababa, respectively. According to the last census survey (2007) projection in 2017, the estimated population of Ginnir, Sinana, and Madda Walabu districts were 159,766, 154,170, and 127,426, respectively. Women of childbearing age numbered 35,357 for the Ginnir district, 34,118 for the Sinana district, and 28,200 for the Madda Walabu district (19). The Ginnir district has 33 kebeles (kebele refers to the smallest administrative unit), of which 30 are rural and 3 are urban; the Sinana district has 29 kebeles, of which 27 are rural and 2 are urban; and the Madda Walabu district has 22 kebeles, of which 20 are rural and 2 are urban. The Ginnir district has eight health centers and 32 health posts, the Sinana district has six health centers and 26 health posts, and the Madda Walabu district has six health centers and 24 health posts. Each district has one hospital in their capital town that serves as referral centers. These hospitals do not provide breast cancer treatment, so women have to travel to Addis Ababa for treatment.

### Patient and public involvement

In the selected household, adult participants responded to the questionnaire.

### Inclusion criteria

All selected women of childbearing age (15–49 years) living in the study area and residing for more than 6 months in the selected district and interest in participating in the study were included in both qualitative and quantitative sections.

### Exclusion criteria

Non-Ethiopians, pregnant women, women with known breast cancer, seriously ill women during data collection, and women not willing to participate in the study were excluded in both quantitative and qualitative studies. Participants in the quantitative study were also excluded from the qualitative study.

### Sample size determination

A single population proportion formula was used to determine the sample size for the quantitative data. A sample size of 841 was calculated based on an estimated proportion of BSE practice of 53.6% (20); the desired precision was 5% with 95% confidence level and a design effect of 2, including a 10% non-response rate.

Six focus group discussions (FGDs) were conducted for the qualitative part, which was supplementary to the quantitative study, with two FGDs in each district. A total of 59 discussants participated (21). Five FGDs had 10 participants in each discussion, whereas 9 participants were involved in one FGD. The inclusion criteria for this study was women aged 15 to 49 years old, with no known breast cancer, and not having other severe illnesses that could have prevented them from responding.

### Sampling procedure

A multi-stage sampling technique was used to select the study participants. In the first stage, three districts were selected randomly. The kebeles in the selected districts were identified and stratified into urban and rural kebeles. The selected urban and rural districts were selected by using a lottery method. The selection of kebeles was dependent on their distance from the capital town of the district, taking the hospital as a center. For the three districts, the furthest kebeles on average were 45 km from the center. Three kebeles closest to the town (at approximately 15 km), three kebeles from the middle region (at the second 15 km), and three kebeles furthest from the town (at the third 15 km) were selected randomly. A list of all households with eligible mothers was identified; finally, we used a sampling frame to pick the study participants using the simple random sampling method.

For the qualitative data, purposive sampling technique was used to select participants. From each selected district, two groups of childbearing-age women not included in the quantitative study were asked to participate in the FGD.

### Data collection and data quality assurance

For quantitative data collection, a standardized questionnaire was initially developed in the English language after reviewing different literature reflecting the purpose of the study (22–24), then translated to the local language (Afan Oromo), and back translated to English. Data were also collected using face-to-face interviews.

Twelve diploma nurses collected interview data guided by three supervisors. The training of data collectors and supervisors mainly focused on issues such as data collection tools, field methods, inclusion–exclusion criteria, and record keeping. The investigators coordinated the interview process and reviewed completed questionnaires on a daily basis to ensure completeness and consistency of the data. The questionnaire was pre-tested on 5% of the sample outside the selected district for this study.

The FGDs were held in the community meeting room and in health posts at the participant’s districts. The participants were women of childbearing age (15–49 years old) not included in the quantitative study and recruited purposively. An interview guide was used to conduct the FGD and a tape recorder was used to capture the conversation. The interview guide was originally prepared in English, then translated to the local language (Afan Oromo), and translated back to English to check the consistency. Each FGD was conducted by two trained female degree holder nurses; one moderated the discussion while the other took notes and recorded the tape. One gatekeeper (non-health professional) for each FGD was assigned.

### Data analysis

The quantitative data was entered into a database using Epi-info, version 3.5.3, and it was exported to SPSS version 20 for data analysis. First, descriptive statistics were used to describe the data. Then, simple logistic regression analysis was carried out; those variables that were significant at *p* ≤ 0.25 were retained for subsequent multivariable logistic regression. Strength of association was tested using adjusted odds ratio (AOR) and 95% confidence interval (CI). The significance level considered for multivariable logistic regressions was *p* ≤ 0.05.

For qualitative data, tape recordings were transcribed, organized in narrative forms congruent with the respondents’ own words, and analyzed under selected themes based on the question guide and summarized manually; i.e., thematic type of data analysis was carried out for the qualitative study.

## Results

### Socio-demographic characteristics

A total of 836 mothers completed the questionnaire for a response rate of 99.4%. The mothers had a mean ( ± SD) age of 31.09 ± 7.34 years. Forty-five percent of the study participants were illiterate. The majority [744 (89.0%)] of the study participants were married. Ninety percent of women were housewives. Regarding the husband’s level of education, more than half [439 (52.5%)] had finished primary school. The majority [463 (55.4%)] of respondents had a monthly income below the poverty line (less than 1,311 EB or $1.90 per month). The majority [475 (56.8%)] of the study participants had a television or radio. The majority [210 (74.9%)] were rural dwellers. Health centers or hospitals were very close to 667 (79.8%) of the study participants (approximately 5 km or less, maximum journey of 2 h on foot) (**Table 1**).

**Table 1:** Socio-demographic characteristics of the respondents in Bale zone, southeast Ethiopia, 2017.

Variable		Number	%
Maternal Age (years)	15–24	146	17.5
	25–34	424	50.7
	35–49	266	31.8
Mother’s Educational Level	Illiterate	376	45.0
	Primary School	362	43.3
	Secondary School	98	11.7
Occupation	Housewife	752	90.0
	Civil Servant	62	7.4
	Merchant	22	2.6
Marital Status	Married	744	89.0
	Separated/Divorced	57	6.8
	Widowed	35	4.2
Husband’s Educational Level	Illiterate	278	33.3
	Primary School	439	52.5
	Secondary School	119	14.2
Having TV or Radio	No	361	43.2
	Yes	475	56.8
Monthly Income	Bellow 1,311	463	55.4
	More than 1,311	373	44.6
Residence	Rural	210	74.9
	Urban	626	25.1
Distance of health facility	≤5 km	667	79.8
	>5km	169	20.2

### Breast self-examination practice

The majority [576 (68.8%)] of the respondents had sufficient knowledge about breast cancer screening. Regarding sources of information, television and radio were the main sources [98 (56.6%)]. This finding was supported by comments from a 19-year-old merchant mother who reported, “…in our place there is no awareness creating activities or education by doctors on breast problem: presence of the disease, its consequences, its sign and symptoms, and its risk factors, and an option for treatment. We heard information from the television and radio. Some of us even heard the presence of its management today from this discussion.”

A middle-aged adult civil servant also stated that, “…I heard my neighbor complaining of a breast disease; I heard also that the disease was cancer. Many women have suffered with disease of the breast, yet I haven’t seen any breast disease on my body…. When I was a child, I heard a neighbor who died due to breast disease. Her breast was wounded, and she was referred and taken far out of this area to receive better treatment in referral hospitals. Even though she had visited many hospitals, she died as the consequence of the disease.”

Only 187 (22.2%) participants knew the appropriate timing to perform BSE. The knowledge of how BSE is performed was known to 491 (58.7%) of the study participants. The majority [650 (77.8%)] of the mothers responded that performing BSE is important. Above 90% of respondents had visited health facilities at some time for sickness. However, only 37% of the mothers had a CBE by health workers during the visit (**Table 2**).

**Table 2:** Breast self-examination knowledge and practice in Bale zone, southeast Ethiopia, 2017.

Variables		Number	%
Have you heard about BSE?	No	663	79.3
	Yes	173	20.7
*If yes, what is/are the source/s?	Health workers	87	50.3
	TV or radio	98	56.6
	Family or friends	55	31.8
Knowledge of BC screening	Not knowledgeable	260	31.2
	Knowledgeable	576	68.8
Knowing how BSE is done	One finger palpation	73	8.7
	Palm and three finger palpation	491	58.7
	Do not know how to do	272	32.6
Ever visited health facility for any sickness	No	76	9.1
	Yes	760	90.9
History of BE by health worker	No	527	63.0
	Yes	309	37.0
Have you practiced BSE?	No	726	86.4
	Yes	110	13.2
Detected abnormalities during BSE (n=110)	Contour	36	32.7
BSE (*n* = 110)	Lump in breast	29	26.4
	Pain of breast	26	23.6
	Itching of the breast	12	10.9
	Tenderness	9	8.2
	No any abnormal	8	7.3
Did you consult health workers? (*n* = 102)	No	38	37.3
	Yes	64	62.7
Is it important to examine	Important	650	77.8
your breast by yourself?	Not important	186	22.2
Knowing appropriate time for	2–3 days after monthly menstruation	187	22.2
performing BSE	Monthly at any time	144	17.5
	Few days before menstruation starts	212	25.4
	Do not know specific time	293	34.9

*Multiple responses allowed; P/E, Physical Examination; HF, Health Facility: health center or hospital.

During another FGD, a housewife in her early 20s reported, “…this breast disease is certainly present in its massive form; I have not experienced this disease on myself; it has hurt many women; and some people say it became “hola” on a woman; some say cancer, and some others say another thing. Even at the moment, there is a woman with breast problem, she gave birth recently, her breast has not produced milk, and she has severe breast ache. The problem is existed among us, however; we don’t know the right time to perform BSE…”

**Figure 1:**
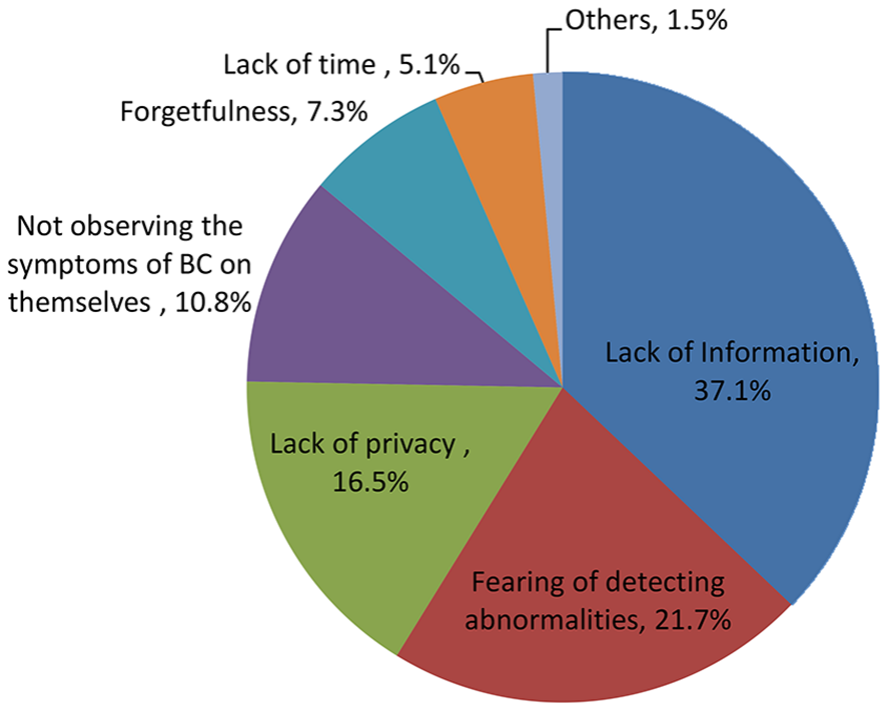
Reasons of non-performing BSE reported by the participants 2017.

In this study, only 110 (13.2%) mothers performed BSE. The main reasons given by the mothers were lack of information [269 (37.1%)], fear of detecting abnormalities [158 (21.7%)], and lack of privacy [138 (16.5%)] (**Figure 1**).

During an FGD about BSE, six mothers stated that BSE was not practiced among them. For example, a civil servant in her early 20s reported that, “…I don’t need to touch and examine my breast if I don’t suspect the problem. If it develops a problem, symptoms force us to touch and examine the breast. It is important to see some internally felt discomfort or externally observed signs and symptoms of disease. We don’t know of breast cancer. Therefore, we don’t give focus to our breast. If we have a previous problem, we check the improvement of that problem from time to time. Unless and otherwise, we don’t examine our breast…”

Those mothers who performed BSE had detected abnormalities in their breasts such as changes in contour [36 (32.7%)] and lump in the breast [29 (26.4%)]. The majority [64 (62.7%)] of the respondents who detected abnormalities in their breasts did consult health workers (**Table 2**).

A middle-aged adult housewife also stated, “… for example, when I have pain of the breast, if all things, even children touch me, I feel pain on both breasts specially at the tip of it, before this time I haven’t ever see such things. For this reason, I have started to touch and look at my breast. If somebody has such problem that woman should touch and examine their own breast, otherwise there is no need to touch…”

During another FDG, a middle-aged adult health extension worker stated that, “…women know their breasts or their bodies especially during a change. Breast pain is not simple, its pain is more severe than other disease; therefore, it is easy to know breast problem on ourselves. However, women have seen this problem traditionally and some of them mostly seek traditional treatment because, they will not permit to expose their breast to health professionals if the disease is not severe. This makes the disease too fatal among our community…”

### Factors associated with breast self-examination practice

Women in the age range of young adults and middle age adults were more likely to practice BSE compared to those women in their early 20s, AOR = 3.61 (95% CI: 1.13, 11.58) and AOR = 9.35 (95% CI: 2.31, 37.85), respectively. Participants who have finished primary education, AOR = 3.88 (95% CI: 1.26, 11.98), and secondary and above, AOR = 11.14 (95% CI: 2.48, 49.96), were more likely to practice BSE than illiterate mothers. In addition, participants who have ever had breast examination previously by health workers were more likely to practice BSE compared to those mothers who have never had CBE, AOR = 3.62 (95% CI: 1.15, 11.45) (**Table 3**).

**Table 3:** Factors associated with breast self-examination practice in Bale zone, southeast Ethiopia, 2017.

Variables		Ever performing BSE		OR (95%) CI	
		No	Yes	COR	AOR
Present maternal age					
15-24		132	14	1.0	1.0
25-34		379	45	1.61(0.76-3.41)	3.61(1.13-11.58) *
35-49		215	51	3.44(1.26-9.39)	9.35(2.31-37.85) *
Mother’s level of education					
Illiterate		357	19	1.0	1.0
Primary school		307	55	2.89(1.37-6.10)	3.88(1.26-11.98) *
Secondary & above		62	36	5.68(2.23-14.51)	11.14(2.48-49.96) *
Occupation	House maker	668	84	1.0	1.0
	Civil servant	52	10	0.57(0.22-1.47)	0.74(0.18-3.12)
	Merchant	6	16	1.83(0.63-5.30)	0.56(0.13-2.46)
Residence	Rural	200	10	1.0	1.0
	Urban	526	100	2.11(0.45-9.03)	3.02(0.98-4.58)
Knowledge of BC screening					
Not knowledgeable		237	23	1.0	1.0
Knowledgeable		489	87	1.84(0.84-4.03)	1.45(0.38-5.53)
Ever visiting health facility for any sickness					
No		32	44	1.0	1.0
Yes		694	66	3.23(1.68-6.19)	0.90(0.31-2.59)
Knowing how BSE is done					
One finger palpation		63	10	1.0	1.0
Palm & 3 finger palpation		407	84	2.47(0.75-8.15)	3.59(0.82-15.79)
Do not know		256	16	0.20(0.06-0.68)	0.36(0.08-1.56)
Having TV or Radio					
No		351	10	1.0	1.0
Yes		375	100	2.35(0.95-5.81)	1.62(0.30-8.64)
History of BE by health workers					
No		508	19	1.0	1.0
Yes		218	91	5.11(2.36-11.06)	3.62(1.15-11.45) *
Have you heard about BSE					
No		621	42	1.0	1.0
Yes		105	68	2.16(1.15-4.05)	1.11(0.41-3.01)
Distance from home to the nearest health facility					
<= 5kms		594	73	1.0	1.0
> 5kms		132	37	0.23(0.09-0.61)	1.42(0.32-6.41)
Importance of BSE					
Important		552	98	1.0	1.0
Not important		174	12	0.35(0.15-0.84)	0.73(0.15-3.58)
Knowing appropriate time for performing BSE					
2-3 days after menses gone		144	43	1.0	1.0
Monthly in fixed days		113	31	0.92(0.37-2.29)	1.50(0.43-5.29)
Before menstruation starts		198	14	0.29(0.11-0.73)	1.05(0.27-4.05)
Do not have specific time		271	22	0.33(0.14-0.76)	0.98(0.26-3.65)

* The test was significant at α = 0.05.

## Discussion

BSE is an important and inexpensive method for the screening and early detection of breast cancer (16, 25). This study showed that 20.7% of the participants responded that they have heard of BSE. This result was very low compared to a study conducted in Malaysia and Libya (26, 27). The mentioned sources of information on BSE for 56.6% of the study participants were the television and radio, altogether. These findings are consistent with a study conducted in Libya (27). Only 13.2% of those polled had used BSE, according to the findings. This result showed that the information was more frequently practiced than studies conducted in Zambia (5%) and south India (2.4%) (28, 29), but less than found in Malaysia (48%), Nigeria (43.5%), and Ethiopia (53.6%) (20, 27, 30). This idea was corroborated by the finding of the qualitative study. The lower BSE practice could be related to the participant’s lack of knowledge, fear of detection of abnormalities, and lack of privacy. This finding was similar to a study conducted in Iran (31).

This study showed that as a mother’s age increases, especially a female in her early 20s, the practice of BSE increases. This is similar to a study conducted in north Ethiopia (20) and two studies conducted in Nigeria by Oladimeji et al. (2015) and Balogun and Owoaje (2003), which revealed that women in their early 20s and middle-aged adult women practiced BSE. This may be due to the fact that at such ages, women are paying more attention to their reproductive activity, resulting in being aware of and caring for their breasts, due to increased contact with health facilities and health professionals than any other period in their lives. These opportunities may expose mothers to information that can be obtained from health professionals to help them to practice BSE.

Different studies showed that practice of BSE was determined by educational level of mothers. A study done in Libya (26) and another study done in Nigeria (30) reported that higher levels of education were significantly associated with BSE practice. Consistent with these previous studies, our study revealed that BSE was related to mother’s level of education. FGD participants reported that they do not know that the right time to perform BSE was one of the major barriers to BSE practice. This is because educated mothers have a greater awareness of the existence of healthcare and benefited from it by using such services. As education empowers women, they have greater confidence and capability of making decisions to use modern healthcare services (14).

In this study, a previous history of breast examination by a health professional was also another predictor for BSE practice. This result was consistent with a study conducted in northwest Ethiopia, which indicated that health extension workers who had previously had their breasts examined by health professionals were a significant predictor of BSE practice. Participants in the FGD noted that there are no effort from health professionals to raise awareness about BSE practice while they are visiting medical facilities. This could be due to the fact that if mothers came to know how breast examination is done, the probability of continuing examination by themselves seems high (22).

### Strengths of the study

We have collected both quantitative and qualitative data, and the questionnaires were produced from standardized sources. The sample size of the study was large enough to establish generalizability to a large population. From the perspective of representativeness, the study included women from three different communities in the rural areas (lowland, temperate, and highland) of Bale Zone.

### Limitations of the study

As a cross-sectional study, a cause-and-effect relationship cannot be established to identify the actual predictor. The study focused only on reproductive-age women and did not include older women, for whom concerns about breast cancer may be greater.

## Conclusion

The study revealed low BSE practice among reproductive-age women, compared with the other studies conducted in Ethiopia. The proportion of the respondents who had ever heard of BSE was also low. Present maternal age, mother’s level of education, and previous history of breast examination by a health professional were found to have significant associations with BSE practices. Thus, increasing maternal education and coverage of breast examination by health professionals is crucial to increase BSE practice.

## Data availability statement

The original contributions presented in the study are included in the article/Supplementary Material. Further inquiries can be directed to the corresponding author.

## Ethics statement

Administrative approval was obtained before conducting the study and an ethical clearance letter was obtained from Madda Walabu University ethical clearance committee with approval number MU-14/51/78. An official letter of collaboration was written to Bale Zone administration and Ginnir, Sinana and Madda Walabu district administrations to get formal permission. Informed consent was obtained from each interviewee. Written informed consent for participation was not required for this study in accordance with the national legislation and the institutional requirements.

## Author contributions

AH, MK, JB, AL and SN developed the concept, developed methods, collected data and analyzed them, and drafted and edited the manuscript. All authors contributed to the article and approved the submitted version.
